# Mutations within the putative protease domain of the human *FAM111B* gene may predict disease severity and poor prognosis: A review of POIKTMP cases

**DOI:** 10.1111/exd.14537

**Published:** 2022-02-13

**Authors:** Afolake Arowolo, Cenza Rhoda, Nonhlanhla Khumalo

**Affiliations:** ^1^ Hair and Skin Research Laboratory Division of Dermatology Groote Schuur Hospital and the Department of Medicine Faculty of Health Sciences University of Cape Town Cape Town South Africa

**Keywords:** *FAM111B*, fibrosis, hereditary fibrosing poikiloderma, myopathy, poikiloderma, POIKTMP

## Abstract

Mutations in the human *FAM111B* gene are associated with a rare, hereditary multi‐systemic fibrosing disease, POIKTMP. To date, there are ten POIKTMP‐associated *FAM111B* gene mutations reported in thirty‐six patients from five families globally. To investigate the clinical significance of these mutations, we summarized individual cases by clinical features and position of the reported *FAM111B* gene mutations as those within and outside the putative protease domain (MWPPD and MOPPD respectively). MWPPD cases had more clinical manifestations than MOPPD (25 versus 18). Although the most common clinical features of poikiloderma, alopecia and hypohidrosis overall occurred in 94%, 86% and 75% of all cases with no significant differences between the MOPPD and MWPPD group, less common features included life‐threatening (pulmonary fibrosis 47% vs. 13%; liver abnormalities specifically cirrhosis 26% vs. 7%) and physically disabling conditions (myopathy 53% vs. 20%; tendon contracture 55% vs. 7%) were more common in MWPPD cases. Similarly, the only 2 cases of POIKTMP with fatal pancreatic cancers were both only in the MWPPD group. This review thus suggests that mutations within the putative protease domain of the FAM111B protein are associated with a broader range of clinical features and may predict increased POIKTMP severity and a poorer prognosis.

## 1 BACKGROUND

Hereditary fibrosing poikiloderma with tendon contractures, myopathy and pulmonary fibrosis (POIKTMP) is a unique multi‐systemic fibrosing and autosomal dominant genetic syndrome. The development of poikiloderma is one of the earliest signs of this disorder.^1^ Consequently, this disease is often misdiagnosed in infants and neonates as Rothmund–Thomson syndrome (RTS), Bloom syndrome, dyskeratosis congenita, Baller–Gerold syndrome, poikiloderma neutropenia, Weary syndrome and Kindler syndrome.^1^

Clinical features in POIKTMP include poikiloderma, myopathy, hypohidrosis, alopecia, tendon/muscle contractures, papules and epidermal atrophy, growth retardation, liver impairment, exocrine pancreatic insufficiency, cataracts and haematological abnormalities.^2^, ^3^, ^4^, ^5^, ^6^ The affected individuals may also experience progressive weakness of proximal and distal muscles.^7^ Furthermore, some patients develop fibrosis of the lungs in later life, causing recurrent bronchitis and abnormal lung function.^3^ Pulmonary fibrosis, however, occurs around the second decade of life and is life‐threatening,^3^, ^4^ with some earlier case reports also mentioning fibrosis of the oesophagus and mediastinal.^2^, ^3^ Furthermore, these POIKTMP cases presented variable phenotypes, some displaying very few clinical features and others displaying up to twenty‐five. We report in this review a total of twenty‐six of these clinical manifestations in thirty‐six cases of POIKTMP (Table 1, Table S1).

**TABLE 1:** Summary of the distribution of POIKTMP cases based on the occurrence rate of all the clinical manifestations associated with the diseases

Clinical phenotypes	No. of cases	% of cases
Poikiloderma	34	94
Eczematous/other skin abnormalities/Blaschko linear pigment/ abnormal pigmentation	33	92
Alopecia	31	86
Hypohidrosis	27	75
Lymphoedema	20	56
Sclerosis of digits/nail dysplasia	15	42
Myopathy/ other muscle abnormalities	14	39
Joint /Tendon contractures	13	36
Restrictive pulmonary function/Pulmonary fibrosis	12	33
Steatorrhea/Exocrine insufficiency	10	28
Growth retardation	10	28
Abnormal liver enzymes	8	22
Liver abnormalities: Hepatomegaly/Cirrhosis/Hepatic encephalopathy	7	19
Other visceral organ abnormalities	8	14
Haematological abnormalities	5	14
Palmoplantar abnormalities	5	14
Bullous lesions	4	11
Absence of tendon reflex	4	11
Eye abnormalities/Cataract	4	11
Cellulitis/ Erysipelas	3	8
Dysphagia	3	8
Erythema	2	6
Delayed puberty	2	6
Pancreatic cancer	2	6
Vasculature abnormalities	1	3
Psychiatric disorders	1	3

Note

No. clinical manifestations = 26; No. of cases = 36. About thirty‐eight clinical features are associated with POIKTMP, which are common or unique to all thirty‐six case reports of POIKTMP. Orange, green and blue coloured rows represent the ‘Common’ (≥40%), ‘Less common’ (≤40% and ≥20%) and ‘Rare’ (≤20%) clinical features occurring in all POIKTMP cases.

The associated or causal missense mutations of POIKTMP were identified in the *Family with sequence similarity 111*‐*member B* (*FAM111B*) gene by whole‐exome sequencing.^3^ Ten POIKTMP‐associated mutations are reported in five families and individuals, totalling thirty‐six cases globally (Table S1). Moreover, three non‐POIKTMP‐associated FAM111B gene mutations are reported in two cases of colorectal cancers and a case of progressive osseous heteroplasia (POH).^8^, ^9^ These mutations/cases do not form part of the ten mutations and thirty‐six cases evaluated in this review.

Although the physiological role of the *FAM111B* gene and gene mutations in POIKTMP are yet to be elucidated, recent studies have suggested that mutations may result in induced cytotoxicity resulting from the upregulation of a putative serine protease‐like domain in the FAM111B protein^10^ or the overexpression of the *FAM111B* gene product as seen in some adenocarcinomas.^11^ Mutations of the *FAM111B* gene are located within and outside this putative protease domain (MWPPD and MOPPD respectively). Most of the reported mutations fall within this protease domain, which spans from position 475–665 of the amino acid sequence of the FAM111B protein. Furthermore, a case report on POIKTMP hypothesized that the location of the mutation could contribute to the phenotypic variation and disease severity^7^; however, this remains unconfirmed.

### 1.1 Objective

To determine whether the location of POIKTMP‐associated *FAM111B* gene mutations with respect to the putative protease domain was associated with disease severity and/or unique clinical features.

## 2 METHODS

The following terms (*FAM111B*, fibrosis, poikiloderma, myopathy, POIKTMP and hereditary fibrosing poikiloderma) were used to search PubMed, and Google Scholar for case reports on POIKTMP from the first description of this syndrome in 2006 till date (i.e. September 2021). We also searched and reviewed other *FAM111B* gene‐related diseases and genome‐wide studies to assist with putting our findings on *FAM111B* gene mutations and POIKTMP case reports into context.

The clinical features in all published cases of POIKTMP were tabulated. Their prevalence was calculated to determine whether there was a difference in the number and types of clinical characteristics between mutations located MWPPD and MOPPD of the FAM111B protein.

## 3 RESULTS AND DISCUSSIONS

### 3.1 Clinical features associated with POIKTMP

Our search identified fourteen case reports of a total of thirty‐six cases of POIKTMP (22 in five families and 14 individual cases). The distribution of these cases and the prevalence of associated clinical features is summarized (Table 1). The skin is the most common and visible manifestation of POIKTMP. Published case reports were clear on some (e.g. poikiloderma (94%), alopecia (86%) and hypohidrosis (75%)) than other manifestations. In particular, the following frequencies were reported for blaschko linear hypo/hyperpigmentation (8%), bullous lesion (11%), dermatitis (3%), eczema (42%), erysipelas (8%), epidermal atrophy/fibrosis (28%), ichthyosis (6%), mottled pigmentation (50%), papules (19%), psoriasis (6%), scleroderma (6%), telangiectasia (11%) and xerosis (11%) (Table S2).

From our summary of these clinical manifestations into twenty‐six groups, we observed that some features are widespread or unique to specific cases (Table 1). In all cases, the most common clinical characteristics (i.e. found in ≥40% of all cases and given the term ‘Common’ in this review) are poikiloderma (94%), eczematous/other skin abnormalities, including blaschko linear pigmentation (92%), alopecia (86%), hypohidrosis (75%), lymphoedema (56%) and sclerosis of digits/nail dysplasia (42%). ‘Less common’ was clinical features that occurred in >20 and ≤40 of all cases, which included myopathy (39%), joint or tendon contractures and restrictive pulmonary function or pulmonary fibrosis was seen in 36 and 33% of cases respectively. Steatorrhea and growth retardation accounted for 28%, with abnormal liver enzymes activities occurring in 22% of all cases. Other clinical features that were classified as ‘Rare’ (appearing in ≤20% of all cases) included liver, visceral, haematological, palmoplantar and eye abnormalities, as well as psychiatric disorders and pancreatic cancer. **Case 6 and 15** (Table S1) were both diagnosed with pancreatic cancer^6^, ^12^ and were the only patients described with POIKTMP and cancer. Two recent studies have associated the *FAM111B* gene's overexpression with lung adenocarcinoma (LUAD); however, the role of *FAM111B* in this cancer of the lungs is unclear.^11^, ^13^ Furthermore, one of these studies suggested that the *FAM111B* gene was highly expressed in LUAD patients and correlated with poor prognosis.^11^ This study further hypothesized that *FAM111B* might participate in the upregulation of tumors via the p53 signalling pathway. A recent genomic study on cancer‐predisposing pathogenic germline variants within homologous recombination repair in patients with advanced cancer revealed mutations in the *FAM111B* gene in two cases of colorectal cancer.^8^ Similarly, a case of progressive osseous heteroplasia (POH), a rare bone disorder characterized by heterotopic ossification of the skin and muscles, was associated with a *de novo FAM111B* gene frameshift mutation in an American 15‐year‐old male.^14^ Although this patient was not diagnosed with POIKTMP, he presented with calcific nodules with a plaque‐like ulcerated skin lesion, contracture and muscle weakness that resulted in patient immobility.^14^ Thus, it will be interesting to investigate the molecular mechanism by which the *FAM111B* gene or mutations contributes to POIKTMP, cancers and POH.

Furthermore, since some case reports are suggestive of the late onset of specific clinic manifestations,^3^, ^4^, ^7^ we compared the clinical features reported in young (<18 years old) and adult (>18 years old) POIKTMP patients (Table S3a,b). From our analysis, fifteen of the 36 cases fell within the first group (i.e. <18 years old), while 21 cases occurred in the latter group. In the ‘Common’ clinical features, early‐onset of poikiloderma and alopecia appears evident in the young versus the adult group (100% vs. 90% and 100% vs. 76%, respectively), as judged by the significant difference (≥10%) in the portion of cases in these groups. This trend was not the case with sclerosis of the digits/nail dysplasia, which saw a higher proportion of patients in the adult group. Similarly, in the ‘Less common’ clinical manifestations, there was an increased distribution of myopathy/other muscle abnormalities (47% vs. 33%), growth retardation (47% vs. 14%) and abnormal liver enzymes (33% vs. 14%) in the young group compared with the adult group. However, the exception was the case with restrictive pulmonary function/pulmonary fibrosis (13% vs. 48%), validating previous observation of the development of pulmonary fibrosis/lung complications in POIKTMP patients from the 2nd to 4^th^ decade of life.^3^, ^4^ There were also higher percentages of young patients in the ‘Rare clinical features’ than the adult group except in the case of pancreatic cancer (0% vs. 10%), suggesting the less common/rare by life‐threatening clinical conditions occur later in adult life (Table S3a,b).

### 3.2 Association of POIKTMP clinical manifestations and position of *FAM111B* gene mutations

Ten POIKTMP‐associated mutations (nine missense and one in‐frame deletion) of the human *FAM111B* gene are reported to date (Table 2). The associated *FAM111B* gene mutation is only available for 31 of 36 (86%) cases, proving that mutations in this gene lead to POIKTMP. Three of the 5 cases with no information on the *FAM111B* gene mutations (i.e. **Cases 2, 21, 22**, Table 2 and Table S1) had similar clinical features and likely had identical mutations with family members who underwent genetic testing.^6^ Furthermore, there was no information on the gene mutations in two individual cases (**Cases 19, 35**, Table 2 and Table S1).^15^, ^16^ In all, we analysed the *FAM111B* gene mutations in thirty‐four cases. Even though, 55% of these mutations resulted from a paternal and maternal inheritance, compared with the 42% of spurious (i.e. de novo) mutations, suggesting this syndrome may also result from a non‐mendelian pattern of inheritance (Table 2).

**TABLE 2:** Analysis of known POIKTMP‐associated *FAM111B* gene mutations in relation to the outside (MOPPD) or within (MWPPD) the putative protease domain and cases of deaths

No.	*FAM111B* mutation	Mutation type	Case no.	Mode of inheritance	Location of mutation (MOPPD/MWPPD	Cases of deaths [No. of deaths]	Cause of death
1	c.1247T>C (p. Phe416Ser)^7^	Missense	30,31	30: Maternal 31: De novo	MOPPD	–	–
2	c.1261_1263delAAG (p. Lys421del)^6^	In‐frame deletion	20–29	Paternal	MOPPD	20,21 [2]	20: No information 21: lung disease
3	c.1289A>C (p. Gln430Pro)^4^, ^5^	Missense	14,32	14: n.d. 32: De novo	MOPPD	14 [1]	Pulmonary fibrosis
4	c.1860T>G (p. Tyr621Asp)^3^	Missense	1–4	1,3,4: Paternal 2: n.d.	MWPPD	2,3 [2]	2: Pulmonary fibrosis 3: Pulmonary fibrosis
5	c.1874C>A (p. Thr625Asn)^4^	Missense	13	De novo	MWPPD	–	
6	c.1873A>C (p. Thr625Pro)^17^	Missense	33	De novo	MWPPD	–	
7	c.1879A>G (p. Arg627Gly)^3^, ^9^, ^12^	Missense	5–8,36	5–6: De novo 7: Paternal 8: n.d. 36: De novo	MWPPD	6, 8^a^,36 [3]	6: Pancreatic adenocarcinoma 8: Unrelated causes (accident)^a^ 36: Decompensated liver cirrhosis
8	c.1883G>A (p. Ser628Asn)^3^, ^4^, ^18^	Missense	9–12	De novo	MWPPD	–	–
9	c.1884T>A (p. Ser628Arg)^1^	Missense	15–18	15: De novo 16–17: Paternal 18: Maternal	MWPPD	15 [1]	Pancreatic adenocarcinoma
10	c.1881 C>T (p. Arg672Ser)^19^	Missense	34	De novo	MOPPD	–	–

^a^ Had liver cirrhosis though died accidentally. Values in parenthesis indicate journal references. n.d., not determined. There is no information on *FAM111B* mutation for Cases 19 and 35. Case 35 also died of pulmonary complications, but there is no information of FAM111B gene mutation.

**FIGURE 1:**
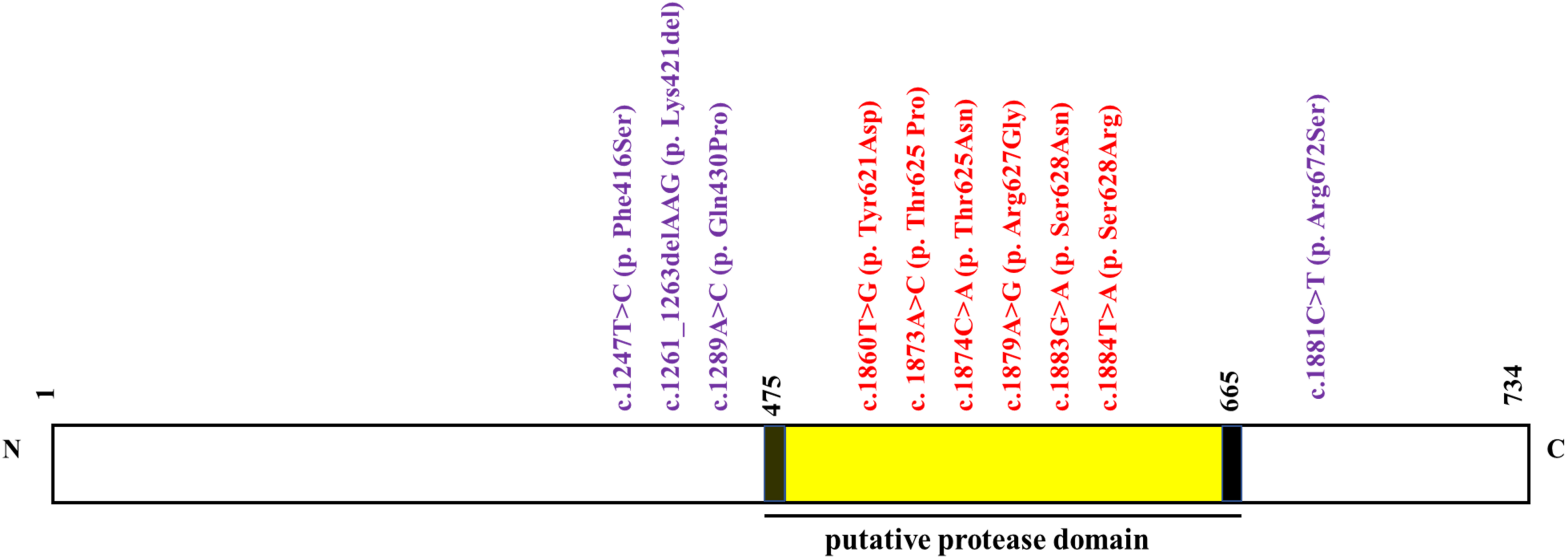
Schematic diagram of the FAM111B protein highlighting the putative protease domain regions (i.e. amino acid residue 475–665, yellow block) and illustrating the clustering of POIKTMP‐associated FAM111B gene mutations outside (MOPPD, indicated in purple text) and mutations within (MWPPD, red text) this putative domain

**FIGURE 2:**
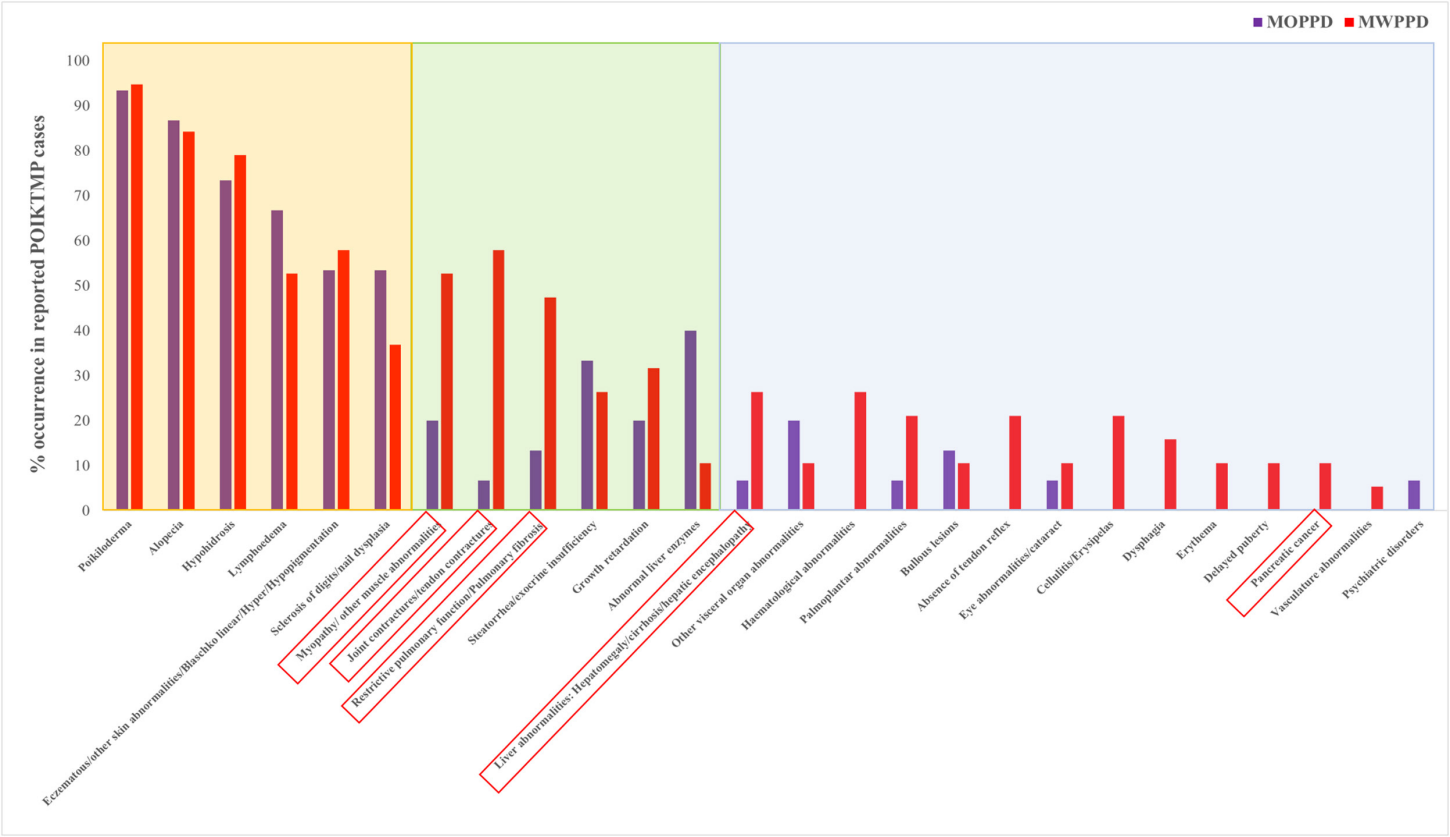
Distribution of POIKTMP‐associated clinical features in patients with MOPPD (purple bars) and MWPPD (red bars) *FAM111B* gene mutations. Orange, green and blue rectangles indicate ‘Common’, ‘Less common’ and ‘Rare’ clinical features respectively. Clinical features outlined in red resulted in deaths and poor quality of life in reported cases

The bioinformatic analysis of the amino acid sequence of the FAM111B protein conducted in our group and elsewhere^10^ identified mutations to cluster either within or outside a putative protease domain which range from position 475–665 (Figure 1). From the ten POIKTMP‐associated *FAM111B* mutations identified to date, four mutations are positioned outside of the putative protease domain (MOPPD), with six located within the putative protease domain (MWPPD) (Figure 1 and Table 2). In these thirty‐four POIKTMP cases, fifteen falls in the MOPPD group, while nineteen are in the MWPPD group (Table S4). Figure 2 shows the distribution of the clinical features of patients with MOPPD and MWPPD. Overall, there were more clinical manifestations in cases with MWPPD (twenty‐five) than MOPPD (eighteen) mutations. The broader spectrum of clinical manifestations could suggest a greater disease burden in the MWPPD group. Interestingly, cases with MOPPD have only one unique clinical feature (i.e. psychiatric disorders) instead of eight (i.e. haematological abnormalities: eosinophilia, platelet counts, absence of tendon reflex, cellulitis/erysipelas, dysphagia, erythema, delayed puberty, vasculature abnormalities and pancreatic cancers) that are exclusive to patients with MWPPD. The distribution of cases based on ‘Common’, ‘Less common’, and ‘Rare’ clinical manifestations as described above, revealed a difference ≤10% in the ‘Common’ clinical manifestations category, which included poikiloderma alopecia and hypohidrosis (Figure 2, orange rectangle) except lymphoedema that occurred in 67% vs. 53%, and sclerosis of digits/nail dysplasia (53% vs. 37%) in cases with MOPPD versus MWPPD respectively. There were substantial differences (≥10%) in the ‘Less common’ category, (elevated liver transaminases (40% vs. 11%), myopathy/other muscle abnormalities (55% vs. 20%), joint/tendon contractures (58% vs. 7%) and restrictive pulmonary function/pulmonary fibrosis (47% vs. 13%), respectively, in MWPPD vs. MOPPD, (Figure 2, green rectangle). Appreciable differences also occurred in the ‘Rare’ category with the proportion of cases high in the MWPPD vs. MOPPD group (e.g. Liver abnormalities: hepatomegaly/cirrhosis/hepatic encephalopathy 26% vs. 7% further suggesting disease severity or increased disease burden in the MWPPD group).

It is noteworthy that the clinical features that contribute to patients’ demise or poor quality of life (i.e. restrictive pulmonary function/pulmonary fibrosis, myopathy/other muscle abnormalities and joint/tendon contractures) and (liver abnormalities: Hepatomegaly/cirrhosis/hepatic encephalopathy) were more pronounced in patients with MWPPD than MOPPD). Even though there was a high proportion of cases with abnormal liver enzymes in the MOPPD group, the cases of liver abnormalities and deaths from this condition were those with liver cirrhosis which was more prominent in the MWPPD group. Similarly, the only two cases diagnosed with POIKTMP and pancreatic cancer, which both died, were in the MWPPD group. Moreover, our overall analysis of the POIKTMP cases revealed no significant differences in the number of deaths resulting from complications of this disease in patients with MOPPD and MWPPD (Table 2).

In all, our analysis tends towards supporting the notion that patients with MWPPD in the *FAM111B* gene have a higher disease burden and poor disease prognosis than patients with MOPPD.

## 4 CONCLUSIONS AND RECOMMENDATIONS

The diagnosis of POIKTMP relies on dermatologic examination and identifying a pathogenic mutation in the *FAM111B* gene. This review highlights the variability of the clinical spectrum of POIKTMP in published cases to date. The three commonest clinical features are early onset of poikiloderma, alopecia and hypohidrosis. Furthermore, these cases’ clinical phenotype differs from other types of hereditary poikiloderma, such as RTS, Werner syndrome, Clericuzio‐type poikiloderma with neutropenia and Kindler syndrome. RTS, diagnosed by mutations of the *RECQL4* gene in affected patients and poikiloderma, is often misdiagnosed due to the similarities in clinical presentations. This misdiagnosis further highlights the importance of screening for *FAM111B* gene mutations in suspected cases of POIKTMP. Also, there are certain less common/rare clinical features (e.g. pulmonary fibrosis and cancer) that occur in these patients in later life.

Furthermore, the mutation location on the *FAM111B* gene may contribute to disease severity and more clinical manifestations in individuals with MWPPD vs. MOPPD. What was surprising was the possibility of pancreatic cancer predisposition in POIKTMP patients with MWPPD. Hopefully, as more cases and further studies are published, the molecular basis and the genotype–phenotype correlation will be elucidated. We, therefore, recommend the careful monitoring of these patients, especially those with MWPPD *FAM111B* mutations, for signs of the less common or rare clinical symptoms of POIKTMP that could exacerbate the already existing poor quality of life or the rate of mortality from this disease.

## CONFLICT OF INTEREST

The authors have no conflict of interest to declare.

## AUTHOR CONTRIBUTIONS

All authors contributed equally to the drafting of this article. AA and NK conceptualized the review. AA and CR searched, reviewed and analysed the data generated from the case reports. AA wrote the first draft of the manuscript. AA and NK revised, edited and organized the manuscript's content.

## Supporting information


**Table S1.** A summary of clinical presentations in documented cases of POIKTMP. Columns highlighted in red indicate patients who died from pulmonary fibrosis or lung disease (orange), pancreatic adenocarcinoma/lung cancer (yellow). Green columns represent patients with liver disease, and patient who died from liver complications (blue).
**Table S2**. A summary of the distribution of an expanded list of skin abnormalities reported in POIKTMP cases.
**Table S3**. A comparison of the clinical features (A) and skin abnormalities (B) reported in young (< 18 years old) and adult (>18 years old) POIKTMP patients.
**Table S4**. A summary of the distribution of POIKTMP‐associated clinical features in patients with MOPPD (purple column) and MWPPD (red column) *FAM111B* mutations. Orange, green and blue rectangles indicate “Common”, “Less common” and “Rare” clinical features respectively.

## Data Availability

The data that support the findings of this study are available from the corresponding author upon reasonable request.
