# Supplementary material for: Mutations within the putative protease domain of the human FAM111B gene may predict disease severity and poor prognosis: A review of POIKTMP cases
Source: Exp Dermatol. 2022 Feb 13;31(5):648–54. doi: 10.1111/exd.14537 (PMC9344908; doi:10.1111/exd.14537)
Supplement: Supplementary file 1 — Table S1. A summary of clinical presentations in documented cases of POIKTMP. Columns highlighted in red indicate patients who died from pulmonary fibrosis or lung disease (orange), pancreatic adenocarcinoma/lung cancer (yellow). Green columns represent patients with liver disease, and patient who died from liver complications (blue). Table S2. A summary of the distribution of an expanded list of skin abnormalities reported in POIKTMP cases. Table S3. A comparison of the clinical features (A) and skin abnormalities (B) reported in young (< 18 years old) and adult (>18 years old) POIKTMP patients. Table S4. A summary of the distribution of POIKTMP‐associated clinical features in patients with MOPPD (purple column) and MWPPD (red column) FAM111B mutations. Orange, green and blue rectangles indicate “Common”, “Less common” and “Rare” clinical features respectively. [file EXD-31-648-s001.doc]

**Supplementary Table 1: A summary of clinical presentations in documented cases of POIKTMP. Columns highlighted in red indicate patients who died from pulmonary fibrosis or lung disease (orange), pancreatic adenocarcinoma/lung cancer (yellow). Green columns represent patients with liver disease, and patient who died from liver complications (blue).**

|  | **Family 1 (2, 3)** | | | | |  | **Family 2 (3, 12)** | |
| --- | --- | --- | --- | --- | --- | --- | --- | --- |
|  | **Case 1** | **Case 2** | | **Case 3** | **Case 4** | **Case 5**  **(3)** | **Case 6** | **Case 7** |
| **General information** |  | |  | | | | | |
| Sex | Female | Male | | Male | Male | Male | Male | Male |
| Age | 26 | Death at 56 | | Death at 30 | 31 | 10 | Death at 32 | 8 months |
| Place of origin | South Africa | South Africa | | South Africa | South Africa | France | Algeria | Algeria |
| Consanguinity | No | No | | No | No | No | Yes | No |
| **Clinical manifestations** |  | |  | | | | | |
| Poikiloderma | Yes | Yes | | Yes | Yes | Yes | Yes | Yes |
| Alopecia | Yes | Yes | | Yes | Yes | Yes | Yes | Yes |
| Hypohidrosis | Yes | Yes | | Yes | Yes | Yes | Yes | Yes |
| Lymphoedema | No | No | | No | No | Yes | No | No |
| Eczematous/Other skin abnormalities/Blaschko linear/Hyper/hypo pigmentation | Yes  Telangiectasia, mottled pigmentation, Papules, Epidermal atrophy/Fibrosis | Yes  Telangiectasia, mottled pigmentation, papules, Epidermal atrophy/Fibrosis | | Yes  Mottled pigmentation,  Papules, Epidermal atrophy/Fibrosis,Scleroderma | Yes  Mottled pigmentation,  Papules, Epidermal atrophy/Fibrosis | Yes  Bullous lesions,  Eczema,  Scleroderma | Yes  Eczema | Yes  Epidermal atrophy/Fibrosis,  Eczema |
| Sclerosis of digits/Nail dysplasia | Yes | - | | - | Yes | No | Yes | No |
| Myopathy/Other muscle abnormalities | Yes | - | | - | - | Yes (adipose infiltration of muscles), amyopathy. | Yes (adipose infiltration of muscles), dystrophy, amyopathy and fibrosis. | No |
| Joint /Tendon contractures | Yes | - | | Yes | No | Yes | Yes | No |
| Restrictive Pulmonary function/Pulmonary fibrosis | Yes | Yes  Died from pulmonary fibrosis | | Yes  Died from pulmonary fibrosis | No | Yes | Yes | - |
| Steatorrhea/Exocrine pancreatic insufficiency | - | - | | - | - | Yes | - | - |
| Growth retardation | No | No | | No | No | No | Yes | No |
| Abnormal liver enzymes | - | - | | - | - | - | - | - |
| Liver abnormalities: Hepatomegaly/  Cirrhosis /Hepatic encephalopathy | No | No | | No | No | No | No | No |
| Other visceral abnormalities | - | No | | Oesophageal fibrosis | - | - | - | - |
| Haematological abnormalities | - | - | | - | - | Yes eosinophilia | No | - |
| Palmoplantar abnormalities | Yes | - | | - | Yes | No | No | No |
| Bullous lesions | No | No | | No | No | Yes | No | No |
| Absence of Tendon reflexes | - | - | | - | - | No | Yes | No |
| Eye abnormalities/Cataract | No | No | | No | No | No | No | No |
| Cellulitis/ Erysipelas | No | No | | No | No | Yes | No | No |
| Dysphagia | No | - | | - | - | No | Yes | No |
| Erythema | - | - | | - | - | - | - | - |
| Delayed puberty | - | - | | - | - | - | Yes | - |
| Pancreatic cancer | No | No | | No | No | No | Yes  Died from intraductal pancreatic adenocarcinoma | No |
| Vasculature abnormalities | - | - | | Elastic degeneration, medial calcification. | - | - | - | - |
| Psychiatric disorders | No | No | | No | No | No | No | - |

N.B. Values in parenthesis are journal references

|  | **Case 8**  **(3)** | **Case 9**  **(3)** | **Case 10**  **(4)** | **Case 11**  **(4)** | **Case 12**  **(4, 17)** | **Case 13**  **(4)** | **Case 14**  **(4)** |
| --- | --- | --- | --- | --- | --- | --- | --- |
| **General information** |  | | | | | | |
| Sex | Female | Female | Female | Female | Male | Male | Male |
| Age | Death at 15* | 9 years | 4 years | 5 years | 27 years | 8 years | Death at 40 years |
| Place of origin | Italy | France/Morocco | France | Ireland | Dominican Republic | France | France |
| Consanguinity | No | No | No | No | No | No | No |
| **Clinical manifestations** |  | | | | | | |
| Poikiloderma | Yes | Yes | Yes | Yes | Yes | Yes | Yes |
| Alopecia | Yes | Yes | Yes | Yes | Yes | Yes | Yes |
| Hypohidrosis | Yes | Yes | Yes | Yes | Yes | No | - |
| Lymphoedema | Yes | Yes | Yes | Yes | Yes | No | Yes |
| Eczematous/Other skin abnormalities/Blaschko linear/Hyper/hypo pigmentation | No | Yes,  Epidermal atrophy/Fibrosis, Eczema, Psoriasis | Yes  Epidermal atrophy/Fibrosis, Eczema, Ichthyosis, Blaschko linear pigmentation | Yes  Bullous lesions,  Eczema, | Yes  Blaschko linear pigmentation | No | Yes  Ichthyosis |
| Sclerosis of digits/Nail dysplasia | Yes | No | No | Yes | Yes | No | No |
| Myopathy/Other muscle abnormalities | Myopathy, amyotrophy, muscle contractures, adipose infiltration, dystrophy and fibrosis. | Myopathy, fibrosis, amyotrophy. | No | Myopathy | Myopathy,  atrophy of muscles, fibroadipose replacement, endomysial fibrosis. | Yes,  Myopathy | Adipose infiltration of skeletal muscle. |
| Joint /Tendon contractures | Yes | Yes | Yes | Yes | Yes | Yes | No |
| Restrictive Pulmonary function/Pulmonary fibrosis | No | Yes | - | Yes | Yes | Yes | Yes  Died of pulmonary fibrosis |
| Steatorrhea/Exocrine pancreatic insufficiency | - | No | Yes | Yes | Yes | No | No |
| Growth retardation | Yes | Yes | Yes | Yes | No | No | Yes |
| Abnormal liver enzymes | - | - | - | - | - | - | - |
| Liver abnormalities: Hepatomegaly/cirrhosis /Hepatic encephalopathy | Yes | No | Yes | Yes | Yes | No | No |
| Other visceral organ abnormalities | - | - | - | - | - | - | - |
| Haematological abnormalities | Eosinophilia | Eosinophilia | No | Eosinophilia | Platelet count | No | No |
| Palmoplantar abnormalities | No | No | Yes.  Palmar erythrosis and palmoplantar keratoderma. | No | No | Palmar erythrosis | Palmoplantar keratoderma |
| Bullous lesions | No | No | No | Yes | No | No | No |
| Absence of Tendon reflexes | Yes | Yes | No | - | Yes | No | No |
| Eye abnormalities/ Cataract | Cataract | No | No | No | Shallow orbits with mild restriction of medial rectus action OU, right macular pigmentary changes | No | Corneal thickness. |
| Cellulitis/ Erysipelas | Yes | No | No | Yes | No | No | No |
| Dysphagia | - | Yes | No | No | Yes | No | No |
| Erythema | - | - | - | - | - | - | - |
| Delayed puberty | Yes | - | - | - | No | - | No |
| Pancreatic cancer | No | No | No | No | No | No | No |
| Vasculature abnormalities | - | - | - | - | - | - | - |
| Psychiatric disorders | No | No | No | No | No | No | Yes, Schizophrenia |

*Died of unrelated causes (i.e., through an accident)

|  | **Family 3 (1, 12)** | | | | |  | **Family 4 (6)** | |
| --- | --- | --- | --- | --- | --- | --- | --- | --- |
|  | **Case 15** | **Case 16** | | **Case 17** | **Case 18** | **Case 19 (15)** | **Case 20** | **Case 21** |
| **General information** |  | | | | | | | |
| Sex | Male | Male | | Female | Female | Male | - | - |
| Age | Died at 64 | 30 | | 27 | - | 13 months | Death at 56^†^ | Death at 67 |
| Place of origin | France | France | | France | France | India | Unknown | Unknown |
| Consanguinity | No | No | | No | No | No | - | - |
| **Clinical manifestations** |  | | | | | | | |
| Poikiloderma | Yes | | Yes | Yes | - | Yes | - | Yes |
| Alopecia | Yes | | No | - | - | Yes | - | Yes |
| Hypohidrosis | Yes | | Yes | Yes | - | Yes | - | Yes |
| Lymphoedema | Yes | | Yes | Yes | - | - | - | Yes |
| Eczematous/Other skin abnormalities/Blaschko linear/Hyper/hypo pigmentation | Yes  Telangiectasia,  Mottled pigmentation, Epidermal atrophy/  Fibrosis, Xerosis, Erysipelas | | Yes  Mottled pigmentation, Epidermal atrophy/  Fibrosis, Xerosis, Erysipelas | Yes  Mottled pigmentation, Epidermal atrophy/Fibrosis, Xerosis, Erysipelas | Yes  Telangiectasia | Yes,  Telangiectasia. Mottled pigmentation, Eczema | Yes  Eczema | Yes  Eczema |
| Sclerosis of digits/Nail dysplasia | - | | Yes | No | - | - | Yes | Yes |
| Myopathy/Other muscle abnormalities | - | | - | Atrophy of thenar and hypothenar eminences. | - | - | - | - |
| Joint /Tendon contractures | Yes | | - | - | - | - | - | - |
| Restrictive Pulmonary function/Pulmonary fibrosis | No | | No | No | - | - | - | Lung disease |
| Steatorrhea/Exocrine pancreatic insufficiency | Yes | | - | - | - | - | - | - |
| Growth retardation | - | | - | - | - | No | - | - |
| Abnormal liver enzymes | - | | - | Increased liver transaminases | - | - | - | - |
| Liver abnormalities: Hepatomegaly/  Cirrhosis /Hepatic encephalopathy | - | | - | - | - | - | - | - |
| Other visceral organ abnormalities | Pancreatic infiltration. | | - | No | - | - | - | - |
| Haematological abnormalities | - | | - | - | - | - | - | - |
| Palmoplantar abnormalities | - | | - | - | - | - | - | - |
| Bulllous lesions | - | | - | - | - | - | - | No |
| Absence of Tendon reflexes | - | | - | - | - | - | - | - |
| Eye abnormalities/Cataract | No | | - | - | - | Keratoconjunctivitis with ectropion. | - | - |
| Cellulitis/ Erysipelas | Yes | | - | - | - | - | - | - |
| Dysphagia | - | | - | - | - | - | - | - |
| Erythema | - | | - | - | Yes | - | - | - |
| Delayed puberty | - | | - | - | - | - | - | - |
| Pancreatic cancer | Yes  Died of Pancreatic adenocarcinoma | | - | - | - | - | - | - |
| Vasculature abnormalities | - | | - | - | - | - | - | - |
| Psychiatric disorders | - | | - | - | - | No | - | - |

†No information on cause of death

|  | **Family 4 (6) (contd.)** | | | | | | | |
| --- | --- | --- | --- | --- | --- | --- | --- | --- |
|  | **Case 22** | **Case 23** | **Case 24** | **Case 25** | **Case 26** | **Case 27** | **Case 28** | **Case 29** |
| **General information** |  | | | | | | | |
| Sex | - | - | - | - | - | - | Female | Female |
| Age | 58 | 57 | 30 | 27 | 28 | 31 | 10 | 8 |
| Place of origin | Unknown | Unknown | Unknown | Unknown | Unknown | Unknown | Unknown | Unknown |
| Consanguinity | - | - | - | - | - | - | - | - |
| **Clinical manifestations** |  | | | | | | | |
| Poikiloderma | Yes | Yes | Yes | Yes | Yes | Yes | Yes | Yes |
| Alopecia | Yes | Yes | Yes | No | Yes | Yes | Yes | Yes |
| Hypohidrosis | Yes | Yes | Yes | Yes | Yes | Yes | Yes | Yes |
| Lymphoedema | Yes | Yes | Yes | Yes | Yes | Yes | Yes | Yes |
| Eczematous/Other skin abnormalities/Blaschko linear/Hyper/hypo pigmentation | Yes  Mottled pigmentation | Yes  Mottled pigmentatio | Yes  Mottled pigmentatio | Yes  Mottled pigmentatio | Yes  Mottled pigmentatio,  Eczema | Yes  Mottled pigmentatio,  Eczema | Yes  Mottled pigmentatio,  Eczema, Bullous lesions | Yes  Mottled pigmentatio,  Eczema |
| Sclerosis of digits/Nail dysplasia | Yes | Yes | No | No | Yes | Yes | No | No |
| Myopathy/Other muscle abnormalities | - | - | - | - | - | - | - | - |
| Joint /Tendon contractures | - | - | - | - | - | - | - | - |
| Restrictive Pulmonary function/Pulmonary fibrosis | - | No | No | No | No | No | No | No |
| Steatorrhea/Exocrine pancreatic insufficiency | Steatorrhea; Low pancreatic isoamylase | - | - | - | Steatorrhea;  Low pancreatic isoamylase | Low pancreatic isoamylase | Low pancreatic isomylase | Steatorrhea;  Low pancreatic isomylase |
| Growth retardation | - | - | - | - | - | - | - | - |
| Abnormal liver enzymes | - | - | - | - | Elevated liver transaminases | Elevated liver transaminases | Elevated liver transaminases | Elevated liver transaminases |
| Liver abnormalities: Hepatomegaly/  Cirrhosis /Hepatic encephalopathy | - | - | - | - | - | - | - | - |
| Other visceral organ abnormalities | - | - | - | - | No | No | Abnormal hepatic biopsy. | Abnormal hepatic biopsy. |
| Haematological abnormalities | - | - | - | - | - | - | - | - |
| Palmoplantar abnormalities | - | - | - | - | - | - | - | - |
| Bulllous lesions | No | No | No | No | No | No | Yes | No |
| Absence of Tendon reflexes | - | - | - | - | - | - | - | - |
| Eye abnormalities/Cataract | - | - | - | - | - | - | - | - |
| Cellulitis/ Erysipelas | - | - | - | - | - | - | - | - |
| Dysphagia | - | - | - | - | - | - | - | - |
| Erythema | - | - | - | - | - | - | - | - |
| Delayed puberty | - | - | - | - | - | - | - | - |
| Pancreatic cancer | - | - | - | - | - | - | - | - |
| Vasculature abnormalities | - | - | - | - | - | - | - | - |
| Psychiatric disorders | - | - | - | - | - | - | - | - |

|  | **Family 5 (7)** | | **Case 32**  **(5)** | **Case 33**  **(18)** | **Case 34**  **(19)** | **Case 35**  **(16)** | **Case 36**  **(9)** |
| --- | --- | --- | --- | --- | --- | --- | --- |
|  | **Case 30** | **Case 31** |  |  |  |  |  |
| **General information** |  | | | | | | |
| Sex | Female | Female | Female | Male | Female | Male | Male |
| Age | 14 months | 24 | 14 | 5 months | 6 | Death at 46 | Death at 17 |
| Place of origin | China | China | Kuwait | China | Mexico | Japan | Italy |
| Consanguinity | No | - | Yes | No | - | - | No |
| **Clinical manifestations** |  | | | | | | |
| Poikiloderma | Yes | Yes | Yes | Yes | Yes | Yes | Yes |
| Alopecia | Yes | Yes | Yes | Yes | Yes | Yes | Yes |
| Hypohidrosis | - | - | Yes | No | Yes | - | - |
| Lymphoedema | - | - | - | No | - | - | Yes |
| Eczematous/Other skin abnormalities/Blaschko linear/Hyper/hypo pigmentation | Yes  Eczema, Psoriasis | Yes  Mottled pigmentation, Blaschko linear pigmentation | Yes,  Papules | Yes  Telangiectasia, Mottled pigmentation, Papules, Eczema, Xerosis | Yes  Papules, Bullous lesions, Eczema | No | Yes  Dermatitis |
| Sclerosis of digits/Nail dysplasia | - | Yes | Yes | - | - | - | - |
| Myopathy/Other muscle abnormalities | No | Yes,  Adipose infiltration | No | No | Yes.  Adipose infiltration | Yes | Yes |
| Joint /Tendon contractures | No | Yes | No | - | - | Yes | - |
| Restrictive Pulmonary function/Pulmonary fibrosis | No | No | No | No | No | Yes  Died of Pulmonary fibrosis. | - |
| Steatorrhea/Exocrine pancreatic insufficiency | - | - | - | No | No | - | - |
| Growth retardation | No | - | Yes | No | Yes | Yes | Yes |
| Abnormal liver enzymes | Elevated alanine and aspartate aminotransferase levels. | - | - | - | Elevated liver transaminases | - | Elevated liver transaminases |
| Liver abnormalities: Hepatomegaly/  Cirrhosis /Hepatic encephalopathy | - | - | - | No | Liver disease.  A biopsy showed lymphocytic ductulitis, duct loss, portal inflammation | Yes | Yes  Died of decompensated liver cirrhosis |
| Other visceral organ abnormalities | - | - | Hypocalcaemia, low vitamin D and mildly impaired renal function. Hypothyroidism | - | - | Peribronchiolar interstitial shadows in the lungs. | - |
| Haematological abnormalities | - | No | . | No | - | - | - |
| Palmoplantar abnormalities | - | - | - | No | - | - | - |
| Bulllous lesions | - | - | - | - | Yes | - | - |
| Absence of Tendon reflexes | - | - | No | No | - | - | - |
| Eye abnormalities/Cataract | - | - | - | - | - | - | - |
| Cellulitis/ Erysipelas | - | - | - | - | - | - | - |
| Dysphagia | - | - | - | - | - | - | - |
| Erythema | - | - | - | Yes | - | - | - |
| Delayed puberty | - | - | - | - | - | - | - |
| Pancreatic cancer | - | - | - | - | - | - | - |
| Vasculature abnormalities | - | - | - | - | - | - | - |
| Psychiatric disorders | No | - | No | No | - | - | - |

**Supplementary Table 2: A summary of the distribution of an expanded list of skin abnormalities reported in POIKTMP cases.**

| **Skin abnormalities** | **No.of Cases** | **% Of**  **Cases** |
| --- | --- | --- |
| Blaschko linear pigmentation  Bullous lesions  Dermatitis  Eczema  Epidermal atrophy/fibrosis  Erysipelas  Ichthyosis  Psoriasis  Scleroderma  Telangiectasia  Xerosis | 3  4  1  15  10  3  2  2  2  4  4 | 8  11  3  42  28  8  6  6  6  11  11 |

**Supplementary Table 3: A comparison of the clinical features (A) and skin abnormalities (B) reported in young (< 18 years old) and adult (>18 years old) POIKTMP patients.**

| **A) Clinical features** | **≤18 years**  **(15 Cases)** | **% Of**  **Cases** | **≥18 years**  **(21 Cases)** | **% Of**  **Cases** |
| --- | --- | --- | --- | --- |
| Poikiloderma  Eczematous/other skin abnormalities/Blaschko linear pigmentation/abnormal pigmentation  Alopecia  Hypohidrosis  Lymphoedema  Sclerosis | 15  13  15  11  8  3 | 100  87  100  73  53  20 | 19  20  16  16  12  12 | 90  95  76  76  57  57 |
| Myopathy/ other muscle abnormalities  Joint /Tendon contractures  Restrictive pulmonary function/Pulmonary fibrosis  Steatorrhea/Exocrine insufficiency  Growth retardation  Abnormal liver enzymes | 7  6  2  5  7  5 | 47  40  13  33  47  33 | 7  7  10  5  3  3 | 33  33  48  24  14  14 |
| Liver abnormalities: Hepatomegaly/Cirrhosis/Hepatic encephalopathy  Other visceral organ abnormalities  Hematological abnormalities  Palmoplantar abnormalities  Bullous lesions  Absence of tendon reflex  Eye abnormalities/Cataract  Cellulitis/ Erysipelas  Dysphagia  Erythema  Delayed puberty  Pancreatic cancer  Vasculature abnormalities  Psychiatric disorders | 5  5  4  2  4  2  2  3  1  1  1  0  0  0 | 33  33  27  13  27  13  13  20  7  7  7  7  0  0 | 2  3  1  3  0  2  2  1  2  1  1  2  1  1 | 10  14  5  14  0  10  10  5  10  5  5  10  5  5 |

| **B) Skin abnormalities** | **<18 years (15 Cases)** | **% Of**  **Cases** | **>18 years**  **(21 cases** | **% Of**  **Cases** |
| --- | --- | --- | --- | --- |
| Skin abnormalities:  Blaschko linear pigmentation  Bullous lesions  Dermatitis  Eczema  Epidermal atrophy/fibrosis  Erysipelas  Ichthyosis  Psoriasis  Sclerodermiform  Telangiectasia  Xerosis | 1  4  1  10  3  0  1  2  1  2  1 | 7  27  7  67  20  0  7  13  7  13  7 | 2  0  0  5  7  3  1  0  1  3  3 | 10  0  0  24  33  14  5  0  5  14  14 |

**Supplementary Table 4: A summary of the distribution of POIKTMP-associated clinical features in patients with MOPPD (purple column) and MWPPD (red column) *FAM111B* mutations. Orange, green and blue rectangles indicate “Common”, “Less common” and “Rare” clinical features respectively.**

| **Clinical features** | **MOPPD**  **(15 Cases)** | | **MWPPD**  **(19 Cases)** | |
| --- | --- | --- | --- | --- |
|  | **No. of Cases** | **% of Cases** | **No. of Cases** | **% of Cases** |
| Poikiloderma  Eczematous/other skin abnormalities/Blaschko linear pigmentation/abnormal pigmentation  Alopecia  Hypohidrosis  Lymphoedema  Sclerosis of digits/nail dysplasia | 14  14  13  11  10  8 | 93  93  87  73  67  53 | 18  18  16  15  10  7 | 95  95  84  79  53  37 |
| Myopathy/ other muscle abnormalities  Haematological abnormalities  Joint /Tendon contractures  Restrictive pulmonary function/Pulmonary fibrosis  Pancreas steatorrhea/exocrine insufficiency  Growth retardation | 3  1  2  5  3  6 | 20  7  13  33  20  40 | 10  11  9  5  6  2 | 53  58  47  26  32  11 |
| Liver abnormalities: Hepatomegaly/Cirrhosis/Hepatic encephalopathy  Other visceral organ abnormalities  Haematological abnormalities  Palmoplantar abnormalities  Bullous lesions  Absence of tendon reflex  Eye abnormalities/Cataract  Cellulitis/ Erysipelas  Dysphagia  Erythema  Delayed puberty  Pancreatic cancer  Vasculature abnormalities  Psychiatric disorders | 1  3  0  1  2  0  1  0  0  0  0  0  0  1 | 7  20  0  7  13  0  7  0  0  0  0  0  0  7 | 5  2  5  4  2  4  2  4  3  2  2  2  1  0 | 26  11  26  21  11  21  11  21  16  11  11  11  5  0 |
